# Progress towards onchocerciasis elimination in Côte d’Ivoire: A geospatial modelling study

**DOI:** 10.1371/journal.pntd.0009091

**Published:** 2021-02-10

**Authors:** Obiora A. Eneanya, Benjamin G. Koudou, Meite Aboulaye, Aba Ange Elvis, Yeo Souleymane, Marie-Madeleine Kouakou, Gary J. Weil, Peter U. Fischer

**Affiliations:** 1 Washington University School of Medicine, Department of Medicine, Infectious Diseases Division, St. Louis, Missouri, United States of America; 2 Centre Suisse de Recherches Scientifiques en Côte d’Ivoire, Research and Development Department, Abidjan, Côte d’Ivoire; 3 UFR Sciences de la Nature, Universite Nangui Abrogoua, Abidjan, Côte d’Ivoire; 4 National Neglected Tropical Diseases Control Program, Ministry of Public Health and Hygiene, Abidjan, Côte d’Ivoire; 5 Ministry of Public Health and Hygiene, Abidjan, Côte d’Ivoire; Faculty of Science, Ain Shams University (ASU), EGYPT

## Abstract

**Background:**

Côte d’Ivoire has had 45 years of intervention for onchocerciasis by vector control (from 1975 to 1991), ivermectin mass drug administration (MDA) (from 1992 to 1994) and community directed treatment with ivermectin (CDTi) from 1995 to the present. We modeled onchocerciasis endemicity during two time periods that correspond to the scale up of vector control and ivermectin distribution, respectively. This analysis illustrates progress towards elimination during these periods, and it has identified potential hotspots areas that are at risk for ongoing transmission.

**Methods and findings:**

The analysis used Ministry of Health skin snip microfilaria (MF) prevalence and intensity data collected between 1975 and 2016. Socio-demographic and environmental factors were incorporated into a predictive, machine learning algorithm to create continuous maps of onchocerciasis endemicity. Overall predicted mean MF prevalence decreased from 51.8% circa 1991 to 3.9% circa 2016. The model predicted infection foci with higher prevalence in the southern region of the country. Predicted mean community MF load (CMFL) decreased from 10.1MF/snip circa 1991 to 0.1MF/snip circa 2016. Again, the model predicts foci with higher Mf densities in the southern region. For assessing model performance, the root mean squared error and R^2^ values were 1.14 and 0.62 respectively for a model trained with data collected prior to 1991, and 1.28 and 0.57 for the model trained with infection survey data collected later, after the introduction of ivermectin. Finally, our models show that proximity to permanent inland bodies of water and altitude were the most informative variables that correlated with onchocerciasis endemicity.

**Conclusion/Significance:**

This study further documents the significant reduction of onchocerciasis infection following widespread use of ivermectin for onchocerciasis control in Côte d’Ivoire. Maps produced predict areas at risk for ongoing infection and transmission. Onchocerciasis might be eliminated in Côte d’Ivoire in the future with a combination of sustained CDTi with high coverage, active surveillance, and close monitoring for persistent infection in previously hyper-endemic areas.

## Author summary

Côte d’Ivoire is endemic for onchocerciasis (also known as “river blindness”). This neglected tropical disease is transmitted by biting black flies that breed in fast flowing rivers. From 1975 to 1991, onchocerciasis control was based on weekly aerial spraying of the insecticide temephos, on black fly breeding sites. Vector control, however, was mostly focused on the northern and central parts of the country. From 1992 to present, mass treatment with ivermectin was implemented in all endemic areas, including forested regions in the south. Here we present the first geospatial estimates of onchocerciasis endemicity over time. Using the machine learning algorithm quantile regression forest, we implemented models to: identify important socio-demographic and environmental factors that correlate with onchocerciasis infection; predict the prevalence and density of infection in areas without ground-truth data; delineate remaining infection hotspots. Our results show that Côte d’Ivoire has made very significant progress in reducing infection parameters over time, and they may help to inform future interventions to achieve the goal of onchocerciasis elimination in Côte d’Ivoire.

## Introduction

Onchocerciasis (“river blindness”) is a neglected tropical disease (NTD) caused by the filarial parasite *Onchocerca volvulus*. It is transmitted through bites of infected black flies of the genus *Simulium*. These flies typically breed in fast-flowing waters, because the high oxygen content of this environment is required for larval development [1]. Infected humans may experience visual impairment that can progress to total blindness. Onchocerciasis can also cause severe dermatitis, skin depigmentation, subcutaneous nodules, epilepsy, and excess mortality [2]. The social consequences of onchocerciasis can be devastating [3].

Côte d’Ivoire is endemic for several NTDs [4]. Efforts to reduce the onchocerciasis burden started in 1975 with vector control activities that were coordinated by the Onchocerciasis Control Programme (OCP) in West Africa [5,6]. Aerial spraying of insecticides was carried out mainly in savannah areas in the northern and central parts of the country where blinding onchocerciasis was prevalent. Since 1992, onchocerciasis control has been largely based on the strategy of administering ivermectin to eligible populations in endemic communities [6], although vector control continued beyond 1992 in some river valleys. Ivermectin distribution was initially piloted by nongovernmental organizations. Between 1995 and 2016 the strategy of community directed treatment with ivermectin (CDTi) was adopted, with initial villages receiving this intervention in 1996. This was provided by the Ministry of Health in collaboration with OCP and the African Programme for Onchocerciasis Control (APOC) that supported ivermectin distribution in countries of Africa that were not covered by OCP. CDTi uses local volunteers to distribute ivermectin in endemic communities. This approach enabled control efforts to be extended to areas that were previously excluded from the aerial spraying programme. APOC coordinated CDTi in some 19 countries that provided an estimated 1 billion ivermectin treatments and prevented some 2 million cases of blindness [7]. APOC ended in 2015, and control efforts for onchocerciasis and other NTDs in the AFRO region of the World Health Organization (WHO) are now coordinated by the Expanded Special Project for Elimination of Neglected Tropical Diseases (ESPEN) [8].

Thus, Côte d’Ivoire has had 45 years of intervention that started with vector control in 1975–1991 that was mostly replaced by ivermectin MDA/CDTi since 1992 [4]. Years of civil unrest (2002 to 2007) interrupted public health interventions [9], and this was especially true in the rebel-held northern regions of the country. However, as onchocerciasis has been eliminated in four countries in the Americas and from several foci in Mali [10], Senegal [10,11], Nigeria [12], Sudan [13,14], and Uganda [15,16], there is increasing evidence that the interventions for onchocerciasis can lead to elimination in some areas and that elimination targets set by the WHO are feasible. It is important, therefore, to identify countries and regions where elimination may be achieved using the current interventions and regions where modified strategies may be needed.

Baseline endemicity is a key determinant that affects the feasibility of onchocerciasis elimination and the time required for treatment with ivermectin [17]. Community microfilarial load (CMFL) is widely regarded as an important measure for categorizing onchocerciasis endemicity [18]. This is a measure of the intensity of infection that is calculated as the geometric mean number of MF per skin snip in adults aged ≥20 years within a community. During early stage of intervention, CMFL is considered a robust measure for determining the true epidemiological situation within an endemic population. However, MF (skin snip) prevalence data is commonly used for classifying endemicity, as only meso- and hyperendemic areas are considered to have a high risk for blinding disease.

Previous modelling studies suggested that either annual or biannual MDA, depending on endemicity, coupled with high levels of therapeutic coverage, should be adequate to achieve the elimination threshold suggested by APOC [19]. Maps that classify infection levels could be useful for identifying areas with the highest risk of infection. They can also identify areas that might be more susceptible to recrudescence of infection following local elimination, either from nearby infection hotspots or from more distant foci within the same transmission zone.

Recent advances in disease prediction provide methods for producing continuous maps with high resolution spatial scales using various modelling approaches [20–24]. These geostatistical models are able to predict infection across a large geographical space using a suite of potential disease drivers such as remotely-sensed climate and environmental data together with relevant socio-demographic data to improve model predictions. Furthermore, continuous maps have been published for a variety of helminth infections [24,25] including vector-borne parasites [20,26] such as lymphatic filariasis [27–30]. O’Hanlon *et al* recently presented a geostatistical map of the pre-control prevalence of onchocerciasis in OCP countries in West Africa [25]. Although that study was a valuable contribution, the ground-truth data used to build the models only considered data from savannah areas, and that excluded important endemic areas in Côte d’Ivoire. Model predictions were extrapolated to the forested southern parts of Côte d’Ivoire where there were no associated survey data. Uncertainty in model predictions increase as one moves farther away from ground-truth data. In addition, data based on MF prevalence (skin snip) alone was used to determine onchocerciasis endemicity. Modeling based on CMFL should provide a more complete picture of the disease burden. Finally, as maps are usually built with high-dimensional satellite data, these data are usually non-linear. Modelling within a machine learning framework can efficiently handle complex relationships between predictor and response variables [22,31].

Therefore, to delineate remaining infection hotspots in Côte d’Ivoire, we modelled the CMFL and MF prevalence prior to and during the use of ivermectin MDA/CDTi. We used a trained quantile regression forest (QRF) model to: i) predict the MF prevalence and intensity of onchocerciasis in unsampled locations at 5 × 5km spatial resolution; ii) identify important socio-demographic and other factors that correlate with onchocerciasis endemicity; and iii) delineate areas at risk of ongoing transmission and potential infection hotspots.

## Methods

### Ethics statement

The process of obtaining ethical approvals, informed consent, and arranging logistical procedures for field surveys were handled in-country by the Ministry of Public Health and Hygiene, Côte d’Ivoire with technical support from WHO. Participants gave verbal informed consent.

### Study areas and onchocerciasis survey data

Côte d’Ivoire is divided into four main ecological zones: savannah in the northern region, pre-forest in the central region, forest in the southern region, and mountainous areas in the west-central region. Blinding onchocerciasis predominantly occurs in the northern region, and early intervention (OCPs use of aerial spraying of vector breeding sites) was focused in eight districts in this region from 1975 to 1991. Following the commencement of mass treatment with ivermectin from 1992, intervention was extended to villages in 53 districts located in the pre-forest and forest regions.

Methods used in the onchocerciasis epidemiological surveys have been previously described [4]. Briefly, proximity to rivers (≤ 5 km) and population size (less than 2000 people) were key determinants that informed the selection of survey villages. Two to four villages were selected for surveys per district depending on accessibility and district size. Selection of survey sites was mainly based on accessibility, although expert knowledge from local health staff and prior onchocerciasis endemicity data were also used to spread survey locations to cover as much geographical space as logistically possible.

For each survey, parasitological examinations included collection of a single skin snip from each posterior iliac crest using a Holth-type corneoscleral punch according to WHO protocols. In sentinel villages where more than one survey was conducted, we chose the infection estimates from the latest survey conducted. The MF load for each person was defined as the arithmetic mean MF count per mg skin.

**Fig 1:**
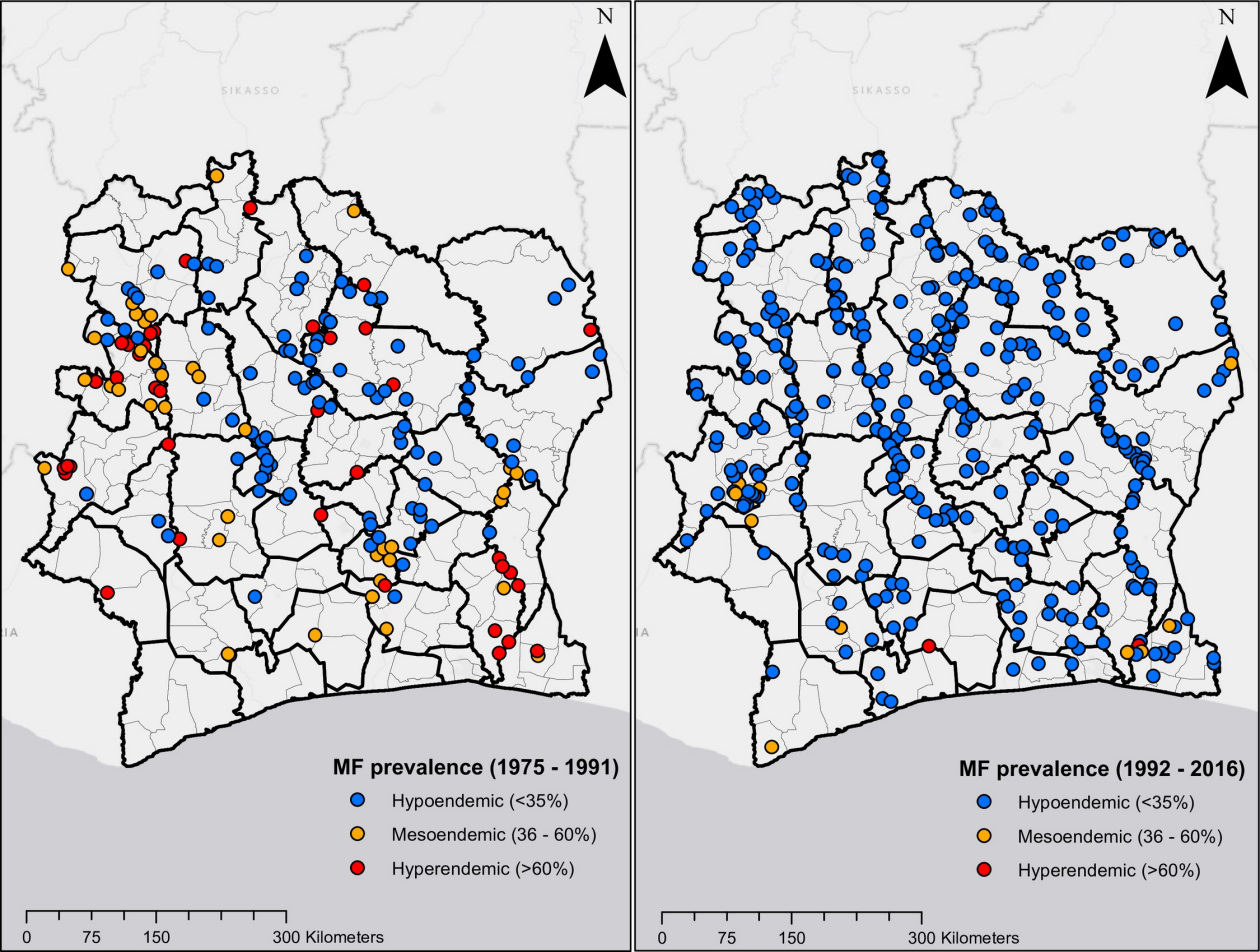
Location of survey sites in Côte d’Ivoire. Plots on the left and right are MF prevalence surveys from 1975 to 1991, and 1992 to 2016, respectively.

**Fig 2:**
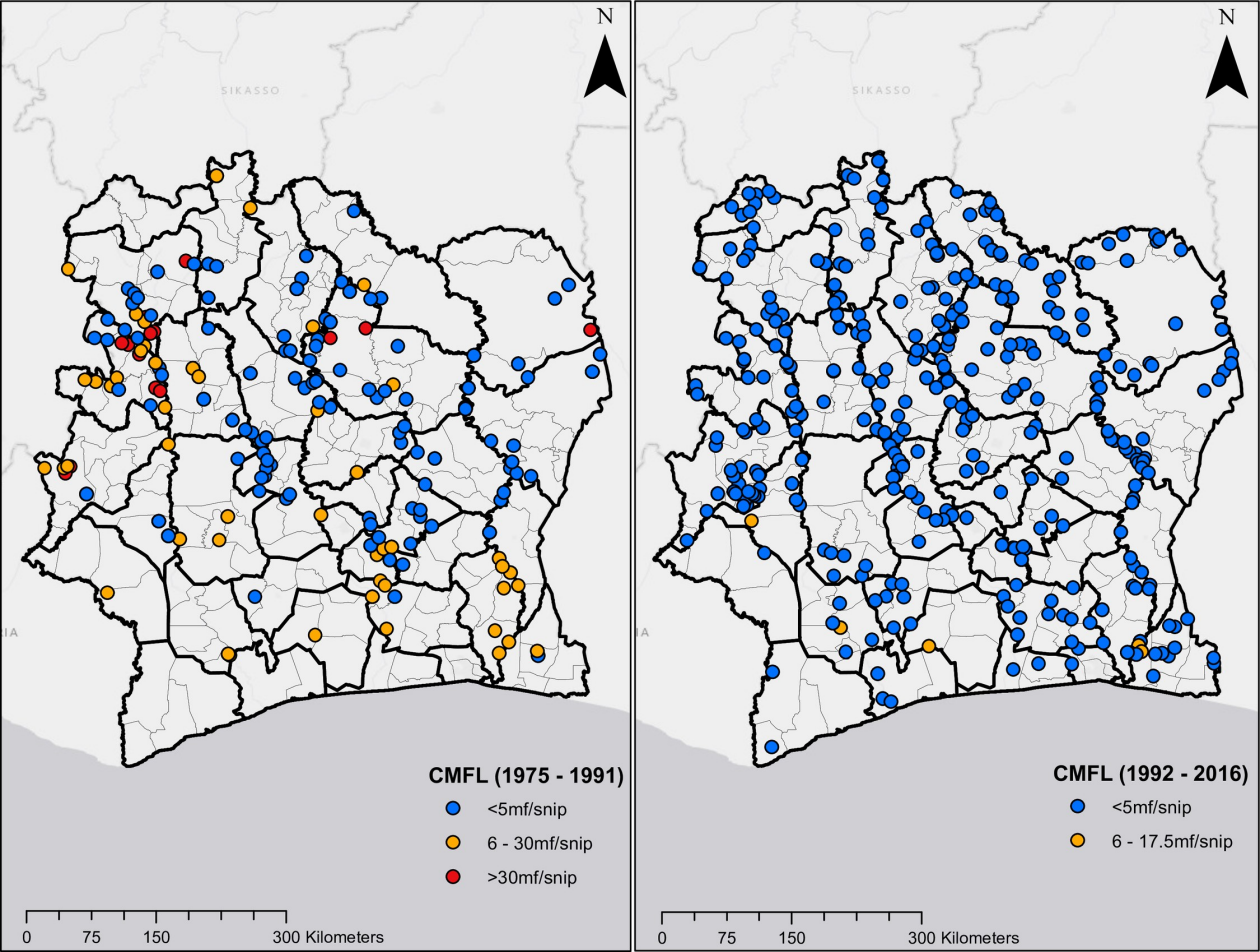
Location of survey sites in Côte d’Ivoire. Plots on the left and right are CMFL surveys from 1975 to 1991, and 1992 to 2016, respectively.

Figs 1 and 2 show the geographical distribution and endemicity level of survey villages, grouped by period.

### Ivermectin distribution

The National Onchocerciasis Control Programme provided ivermectin MDA in about 1500 villages annually between 1992 and 2002. Residents >5 years of age in eligible communities (i.e. MF prevalence of ≥35% or CMFL of ≥ 5MF/snip) were targeted. Pregnant women and persons with severe illnesses were excluded. Communities located <5 km from a river with populations of about 2000 were prioritized for treatment. From 1995, APOC’s CDTi approach was adopted for ivermectin distribution [6]. The coverage goal of the CDTi programme was to treat 65% of the population in endemic villages. Since 2015, the onchocerciasis CDTi program has been integrated with MDA that provides ivermectin plus albendazole annually for elimination of lymphatic filariasis in co-endemic areas.

### Socio-demographic, climatic and environmental data

Interpolated climate and remote sensing data used in combination with statistical models to map the distribution of diseases as well as other health indices has accelerated greatly in the past decade. These data layers are created by collating large amounts of point-level data from weather stations globally. Smoothing algorithms are used to produce continuous maps. We downloaded climate variables related to temperature from the WorldClim database [32]. Here climate layers are presented as smooth maps of mean monthly climate data obtained for the period 1950–2000 from thousands of weather stations globally (data from ~15,000 weather stations were used to estimate the minimum and maximum temperature variables).

Access to communities was a key determinant for selecting survey sites for onchocerciasis surveys. In order to account for this in our analysis, we processed data from the WorldPop repository [33,34] that measures remoteness and proximity to human settlements. Variables such as travel time to nearest large settlement, proximity to major roads and night-time lights were considered. Furthermore, gridded population density estimates, Euclidean distance to permanent inland water bodies and the ocean, terrain slope and altitude were all downloaded from the WorldPop repository. Vegetation cover types (according to the United Nations land cover classification system) were extracted from the GlobCover project at the European Space Agency [35]. Here maps are derived by an automatic and regionally-tuned classification of a 300m medium resolution imaging spectrometer (MERIS) sensor on the ENVISAT satellite mission.

Because onchocerciasis is known to be endemic in agricultural communities, we considered covariates such as designated croplands and areas with established irrigation infrastructure. These data layers were downloaded from the Global Map of Irrigation Areas of Food and Agricultural Organization (FAO) [36]. Here, a digital map of irrigation areas was developed by collating over 10,000 sub-national irrigation records from census surveys and reports available to the FAO and World Bank to create geo-spatial layer for irrigation density (defined as continuous grid-cells that are equipped for irrigation) [37]. Finally, data for household wealth and maternal education were downloaded from the Socioeconomic Data and Applications Center, Columbia University [38], and data on housing type were obtained from the Malaria Atlas Project, University of Oxford [39]. Here housing type was categorized as either being built with finished materials (e.g. cement, bricks, or tiles) or built with natural or unfinished materials (e.g. earth, sand, or palm flooring) [23]. These variables were considered as proxies for wealth.

Changes in the covariates over time were assumed to be negligible for this analysis. For example, the WorldClim dataset uses interpolated data averaged over a 50-year period. Although this dataset pre-dates some of the epidemiological surveys in our study, we assumed that changes that may have occurred in this covariate have been negligible and that it accurately reflects climatic conditions during the study period. Other covariates considered in our model were modelled estimates, and we treated any temporal trends that may exist as negligible. Table 1 shows a list of the covariates and their sources.

All input grids were resampled to a common spatial resolution using the nearest-neighbor algorithm [40] and then clipped to align to the geographical boundaries of Côte d’Ivoire. Raster manipulation and processing were done using the *raster* package in R [41].

**Table 1:** Environmental variables used in analysis and their sources.

Variables	Source
Distance to permanent inland water bodies (rivers and streams)	WorldPop [33]
Distance to coastline	
Distance to major roads	
Night-time lights	
Population density	
Slope of terrain	
Altitude	
Travel time to cities	Malaria Atlas Project [23,39]
Housing type	
Cropland	Food and Agriculture Organization of the United Nations [36,42]
Irrigation	
Vegetation cover	European Space Agency [35]
Household wealth	Socioeconomic Data and Applications Center [38]
Maternal education	
Average minimum temperature	WorldClim [32]
Average maximum temperature	

### Building the model

#### Selection of socio-demographic and environmental covariates

In preparing our dataset for analysis, we extracted values of the covariate raster layers that corresponded with survey locations in Côte d’Ivoire. We considered an initial set of 16 covariates. It is standard practice, however, to account for as much variation in the covariates as possible before building a spatial model. Therefore, within a non-spatial framework, we explored a two-step procedure for covariate selection.

First, to assess for the presence of multicollinearity in our set of covariates, we computed the variance inflation factor (VIF) [43]. Excluding covariates with high VIF values ensures that retained covariates are statistically independent, and reduce variance in models. We set the VIF value as 10, which is a generally accepted threshold [43]. From this selection stage, six covariates (proximity to major roads, night-time lights, cropland, irrigation, maternal education and slope of terrain) were excluded from further analyses. As observed MF prevalence and CMFL values in our dataset were highly correlated (Pearson’s correlation = 0.872), for the purposes of identifying the relationship between predictor and response variable, we defined our response variable (infection status) as binomial.

Second, we assessed the relative importance of the covariates to the response variable. For this, we modelled using a boosted regression tree (BRT) algorithm [44]. The BRT produces an additive regression model in which trees are fitted in a forward, stepwise fashion [44]. To compute the relative importance of the covariates to the response variable, the frequency of the selection of covariates for splitting, weighted by the squared improvements to the model and averaged over all trees are calculated. Higher variable relative importance values, computed as percentages, indicate greater contribution to the model. Variables with no substantial contribution (relative importance threshold set as 10%) were excluded from further analysis. Vegetation cover and population density variables were dropped at this stage. The remaining eight covariates: proximity to permanent rivers and streams, altitude, proximity to coastline, household wealth, average minimum and maximum temperature, housing type, and travel time to major cities, were included in the final analysis. Covariate selection was performed using the *vif* and *gbm* packages in R [41].

#### Quantile regression forest (QRF) algorithm

The QRF is an ensemble learning algorithm for classification and regression based on the construction of decision trees. It efficiently handles large, complex and multi-dimensional satellite data [45]. Studies have shown that this algorithm outperforms traditional regression models under similar modelling scenarios [31,46].

Briefly, trees are grown through recursive binary splits from a primary root node which contains all response and explanatory data. For each split, a new root node is grown using a random subset of approximately one-third of the data. Therefore, each partition contains a random bootstrapped sample of two-thirds of the dataset. The bootstrapped dataset uses a process known as ‘bagging’, whereby resampling is done with replacement, and that prevents model overfitting. Unlike random forest (RF) models that consider mean values of the sample of response variable at each splitting node, the QRF model considers the complete range of values in the response variable for splitting. This process enables a more rigorous measure of uncertainty and quantile determination [45]. The splitting process is repeated until a terminal node is reached. The average of all the trees is then computed and used to make predictions. During the splitting process, variables that were not selected, known as ‘out-of-bag’ cases, are used to conduct internal cross-validation to assess the predictive performance of the model and to generate estimates of the relative importance of explanatory variables.

#### Model implementation and performance measures

We performed a variogram analysis in order to explore the spatial autocorrelation in observed data. This is an exploratory tool widely used in geostatistics [47]. It gives a measure of the variability between pairs of geo-referenced outcome data points (in this study MF prevalence and CMFL). A variable importance analysis was computed to identify the most relevant predictors for onchocerciasis infection. These predictors are ranked in order of contribution to the model for predicting infection in unsampled locations [48]. To explore the relationship between the suite of predictors used in the model building and observed onchocerciasis infection data, we produced marginal effects plots.

We then used a QRF model [45] to map MF prevalence and intensity of onchocerciasis for Côte d’Ivoire for two time periods, namely the vector control period (1975 to 1991) and ivermectin MDA/CDTi period (1992 to 2016). We first fitted an RF model to tune parameters for use in the QRF model. This process informs the optimum number of explanatory variables to be considered at each recursive node split in the QRF model. In building the QRF model, for each directly modelled response variable (MF prevalence and CMFL), data were partitioned to retain a random subset of 30% of data points for validation, while the model was trained on the remaining 70%. A 10-fold internal cross-validation on out-of-bag data was computed and repeated five times. Model evaluation for each response variable was presented as the root mean squared error (RMSE) and R-squared (R^2^) values. Variable importance, estimated using the out-of-bag data from the internal cross-validation, was presented as percentage increase in mean square error. The Pearson’s correlation coefficient was calculated between pairs of observed and predicted values. Final model predictions were presented as mean values projected at a spatial resolution of 5 × 5 km^2^. Uncertainty estimates were presented as standard deviations.

As the QRF algorithm is not a spatially explicit model, we included the geographical coordinates of the observed data to account for the effects of spatial heterogeneity of survey locations in our model predictions [49] in addition to the spatial structure of covariates,. The RF and QRF models were implemented using the *randomForest* and *quantregForest* packages in R [41]. Raster maps of predictions and uncertainty were exported into ArcGIS [50] for final visualization.

## Results

### Variogram analysis

**Fig 3:**
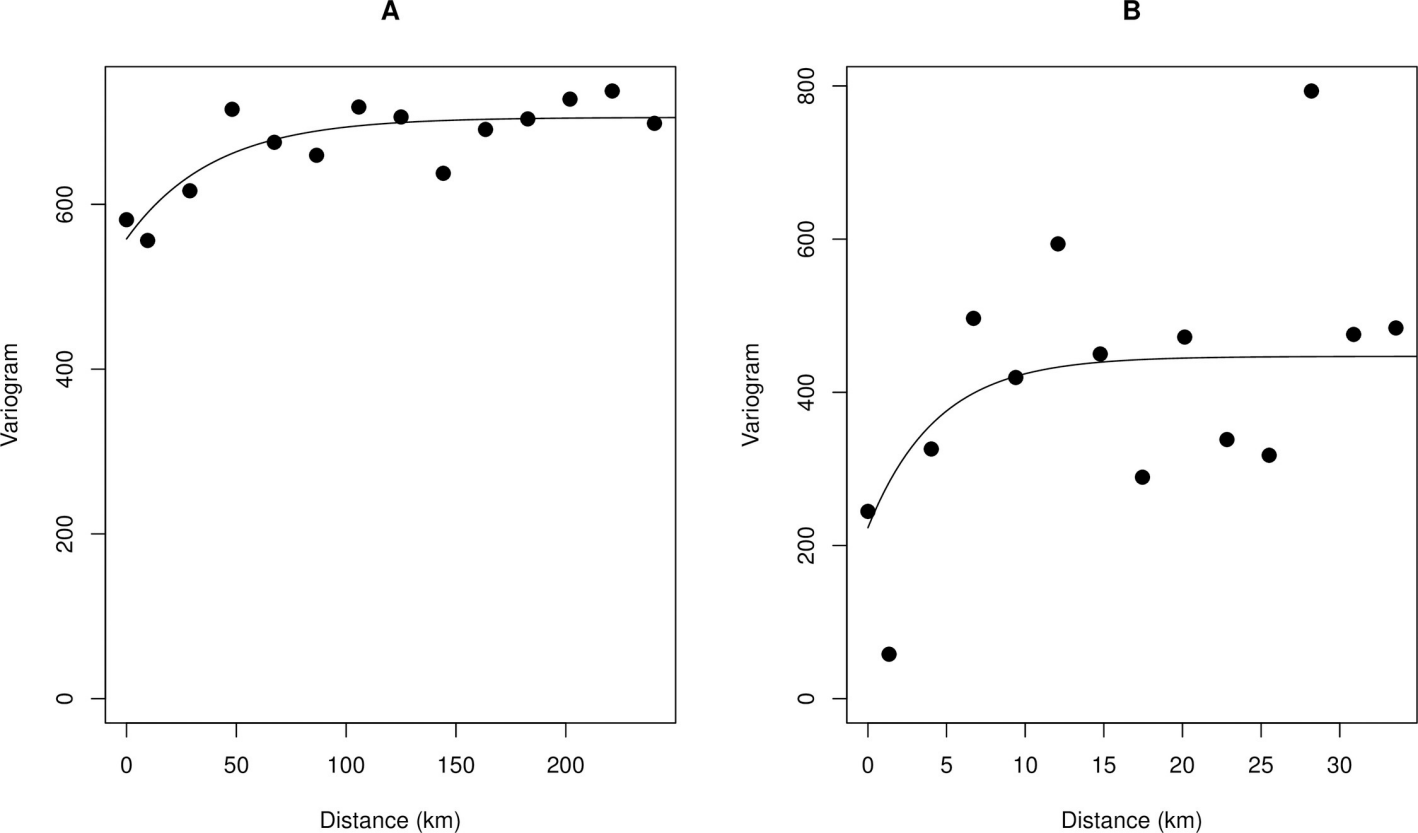
Variogram plot showing the spatial autocorrelation in observed onchocerciasis infection data. **A.** MF prevalence **B.** CMFL. The empirical variogram is represented by the black dots; the theoretical variogram is represented by the solid black line.

Fig 3 shows that there is significant spatial autocorrelation in the observed MF prevalence data, although spatial autocorrelation starts to decay beyond 250 km. In contrast, there was limited spatial autocorrelation for CMFL, even at shorter distances.

### Variable importance analysis

**Fig 4:**
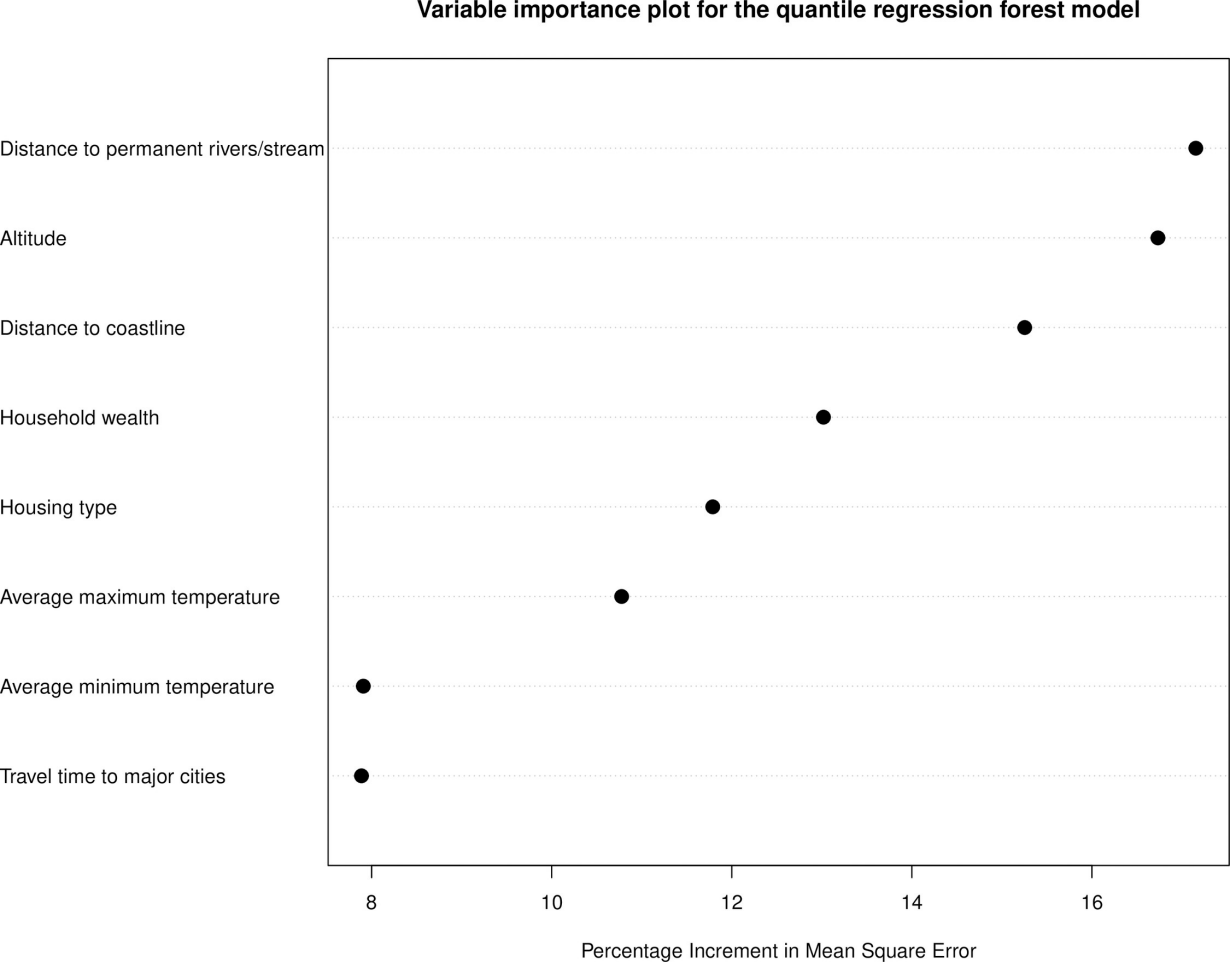
Variable importance for onchocerciasis infection in the trained quantile regression forest (QRF) model.

Fig 4 is a plot of the percentage increment in mean square error computed for the final suite of variables included in the trained QRF model. The most important predictors were proximity to permanent rivers and streams, proximity to the coast, altitude and household wealth.

### Marginal effects plots of covariates included in the model

**Fig 5:**
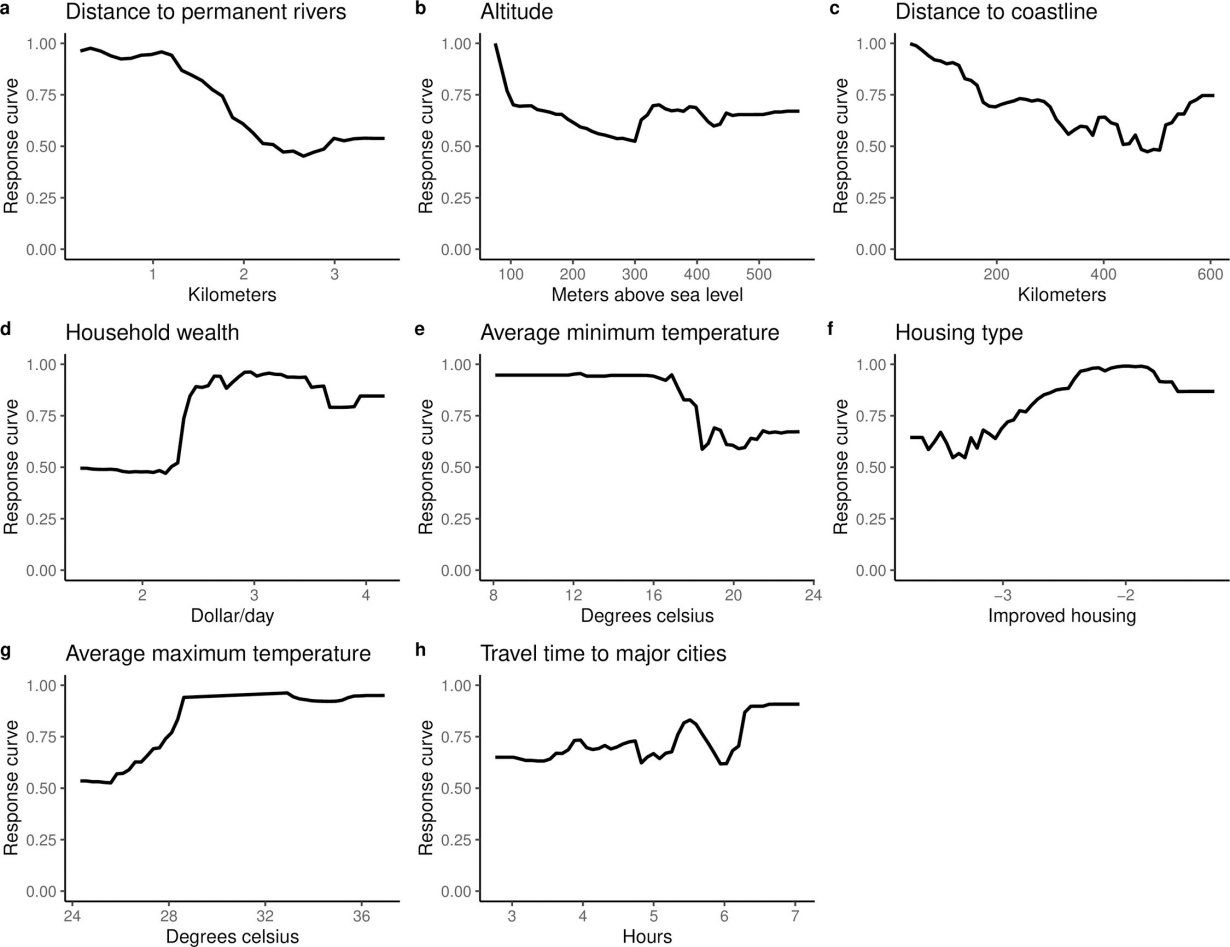
Marginal effects plots for covariates included in the QRF model. The Y-axis is the response (probability of onchocerciasis infection) and the X-axis is the covariate values.

Fig 5 indicate that the probability of onchocerciasis infections decreases with increasing distance from rivers and streams and the coast. Also, higher household wealth and better housing type (houses built with modern materials) were positively correlated with onchocerciasis infection. Travel time to major cities had little effect on onchocerciasis infection.

### MF prevalence and CMFL circa 1991 and 2016

The overall mean observed MF prevalence decreased from 43.2% circa 1991 to 7.6% circa 2016, and the observed mean CMFL decreased from 13.8 MF/snip circa 1991 to 0.6 MF/snip circa 2016. The relatively greater decrease in CMFL means that ivermectin had a greater effect on infection intensity than on prevalence.

**Fig 6:**
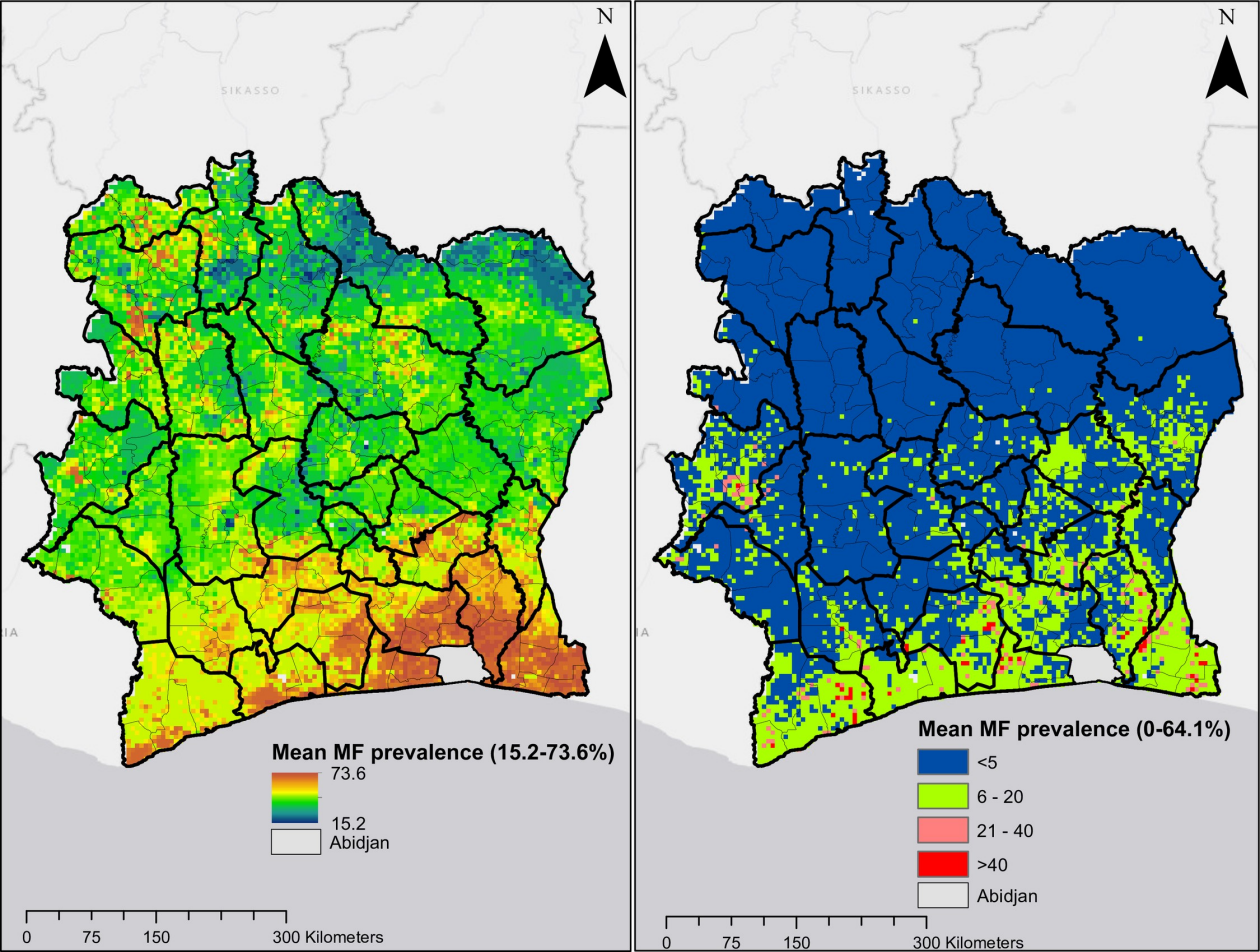
Predicted mean MF prevalence. Plot on the left and right are predictions for onchocerciasis endemicity for circa 1991 and circa 2016, respectively.

Maps presented in Fig 6 show predicted MF prevalence circa 1991 and circa 2016. These maps indicate that prior to mass treatment with ivermectin, all regions in Côte d’Ivoire were endemic for onchocerciasis, although areas in the north were hypo-endemic and south were generally meso- to hyper-endemic. Following the use of ivermectin, endemicity levels decreased significantly throughout the country. However, infection persists in focal areas in the south and central regions. The overall predicted mean MF prevalence decreased from 51.8% circa 1991 to 3.9% circa 2016. The mean Pearson’s correlation between observed and predicted MF prevalence was 0.687 for model trained with surveys conducted from 1975 to 1991and 0.632 for model trained with surveys conducted from 1992 to 2016.

**Fig 7:**
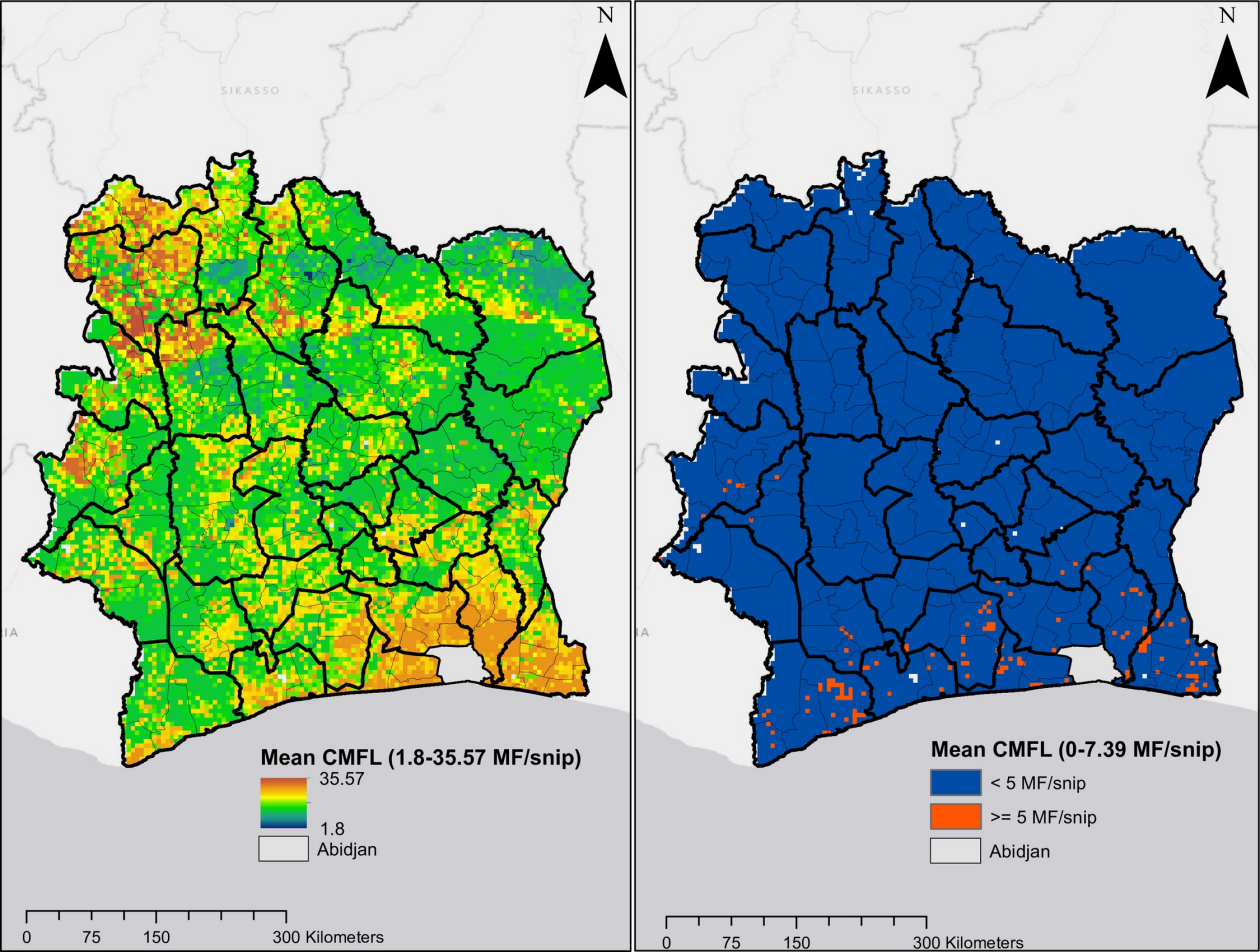
Predicted mean CMFL. Plot on the left and right are predictions for onchocerciasis endemicity for circa 1991 and circa 2016, respectively.

Predicted CMFL data shown in Fig 7 also indicate that onchocerciasis infection was widespread in Côte d’Ivoire in the pre-ivermectin, although values were higher in the northern and southern regions of the country. The overall predicted mean CMFL was 10.1 MF/snip circa 1991. This decreased dramatically following the use of ivermectin to 0.1 MF/snip circa 2016, when CMFL predictions show no infection in most pixels in the map, although ongoing infections were predicted in areas in the south and west-central regions. The mean Pearson’s correlation between observed and predicted CMFL in surveys conducted prior to ivermectin MDA/CDTi was 0.664, and 0.583 for surveys conducted during CDTi.

The RMSE and R^2^ values for the trained QRF model were 1.14 and 0.62 respectively for the model trained with onchocerciasis data from 1975 to 1991. The RMSE and R^2^ values for the model trained with infection data collected from 1992 to 2016 was 1.28 and 0.57, respectively.

## Discussion

In this study, we have produced continuous maps of onchocerciasis MF prevalence and CMFL in Côte d’Ivoire circa 1991 and 2016. We have also identified important socio-economic and environmental variables that correlate with onchocerciasis infection. Our maps illustrate significant reductions in both MF prevalence and CMFL that occurred after the introduction of ivermectin in 1992. We have also identified potential remaining infection hotspots, mainly in the southern region of Côte d’Ivoire. We believe that the data presented in this study are useful for understanding changes in the spatial distribution of onchocerciasis in Côte d’Ivoire over time.

The predictive accuracy of machine learning models is usually assessed by exploring the ability of models to correctly predict results for an independent dataset [51]. As there was no independent dataset available, we trained the QRF model on a random sample of 70% of the total data and predicted results for the held-out 30%. Predictive accuracy was presented as R^2^, which is the percentage of variation explained by the covariates in our model. The model trained with data collected before and during the ivermectin era had R^2^ values of 62% and 57%, respectively. Although these values are reasonably high compared to those presented in other modelling exercises [28], we believe that our model would have performed even better if we had been able to incorporate more detailed information on onchocerciasis morbidity and interventions (vector control and ivermectin treatment) as predictors in our model. This is also shown in our uncertainty maps, presented as standard deviations around per-grid square predictions (S1 Fig). Where standard deviations are high, confidence in model predictions is lower than in locations where the standard deviation is low. Here the contrasting uncertainty maps in the two periods studied may reflect missing intervention data; vector control (mostly in the northern and central regions from 1975 to 1991) and ivermectin treatment in the southern region that started in 1992.

Migration of humans and the drifting of black flies by monsoon winds from far distant hotspots with ongoing infection can also lead to resurgence of infection in areas previously cleared [52–55]. Our variogram analysis shows that the spatial autocorrelation in the observed MF prevalence data has a range of ~250 km (Fig 3A). This is consistent with previous findings [25] and with reports that black flies can travel and infect humans several hundred kilometers from their breeding sites [55–57], although biting density drops to 10% of its highest levels in areas that are more than 5 km from breeding sites [58]. However, if infections levels in humans are reduced to elimination thresholds due to effective intervention measures, resurgence is unlikely regardless of local increases in vector populations.

Although machine learning algorithms are increasingly being used for spatial modelling, they sometimes fail to account for the spatial structure of the outcome of interest. In order to correct for this, in addition to using spatially referenced predictors, we included the geographical coordinates of survey villages in our dataset as covariates in our final model. This adds spatial structure and improves model predictions, as previously reported [49]. We extended the methods of Eneanya *et al*. [27] to carry out a robust, step-wise selection procedure to identify the best suite of uncorrelated explanatory variables. Multicollinearity often arises in statistical models, and it can lead to unstable estimates of the variance of regression coefficients [43].

Remotely-sensed covariates that are associated with the modeled outcome aid in defining the natural geographical limits of the prediction. This improves the ability of the model to explain the variability in predicted outcomes and to account for further spatial structure during the modelling process. We considered land areas designated as cropland, the presence of irrigation infrastructure, type of housing, and household wealth as covariates for infection. These have not been used as predictors in previous spatial models for onchocerciasis. However, we view these as key determinants of the potential distribution of onchocerciasis for the following reasons; i) Agricultural communities are historically known to be highly endemic for onchocerciasis. Farms in these communities are often situated near rivers that provide water for crops. As black flies bite outdoors, farmers and others working outdoors have an increased risk of infection. ii) Wealthier households are more likely to have land for farming.

Our model predicts meso- to hyperendemic hotspots in areas of northern Côte d’Ivoire prior to ivermectin treatment. Previous predictions for these areas [25] were generally higher than ours. In their work, O’Hanlon *et al*. only considered villages in the OCP region that were intervention naïve, whereas villages in our dataset for this area in Côte d’Ivoire had received multiple years of intervention by vector control. Our model also predicted high-level endemicity in the southern part of the country, and those predictions are consistent with results from studies such as that of Dadzie *et al*. [59]. The authors documented high onchocerciasis prevalence in 11 first-line villages in the lower areas near the rivers Bandama and Comoe. Similarly, known hyperendemicity in the Cavally River valley in the western region [60] was also captured in our model.

Our study confirmed that onchocerciasis infection is negatively associated with increasing distance from rivers. Elevation and proximity to breeding sites were reported to be important predictors of onchocerciasis endemicity and severity in Cameroon [61] and Venezuela [62]. Temperature, an important predictor in our model, is linked to development of the black fly vector and to development of parasite larvae in the vector [63]. Proximity to major cities was the least important variable in our model, perhaps due to the extensive geographical coverage of the survey dataset used to construct the models.

Our study predicted high onchocerciasis prevalence for much of southern Côte d’Ivoire. The native vegetation type in this area is rain forest, and high prevalences have been observed for forest-type onchocerciasis in Côte d’Ivoire [64] and elsewhere [65]. Furthermore, data presented by Adjami *et al*. suggest that the savanna-dwelling species *S*. *damnosum s*.*s*. appear to thrive in areas along the middle Bandama river leading to an expansion of savanna type *O*. *volvulus* [66]. This may be as a result of climatic and anthropogenic changes that have resulted in large scale deforestation in West Africa.

Ground-truth data for villages to the east and west of Abidjan indicated meso- or hyperendemic. A recent meta-analysis of onchocerciasis-induced epilepsy in West Africa recorded cases in the south of Côte d’Ivoire [67], in keeping with our model predictions for this area. As there are no ground-truth data for greater Abidjan city, our model made predictions based on these nearby villages and corresponding environmental limits of the covariates. This explains why our maps predict that areas in greater Abidjan were hyperendemic prior to treatment with ivermectin. Therefore, our model may be missing some important covariates that are peculiar to the commercial capital city of Abidjan. As it is known that onchocerciasis is not endemic in Abidjan, we have excluded Abidjan from our final prediction maps. However, it might be useful to perform field surveys to verify the absence of onchocerciasis infection and onchocerciasis-induced epilepsy in the Greater Abidjan area.

Data used to build our predictive spatial models were from surveys conducted by the National Onchocerciasis Control Programme. One limitation of these data is that surveys were not conducted at random; thus, villages surveyed may be skewed towards areas with known high endemicity. In addition, the original programme areas were selected using entomological knowledge about vector breeding sites and information on the distribution of clinical onchocerciasis in villages. Despite these limitations, data from the National OCP comprises an extensive source of standardized parasitological survey data that cover large areas within Côte d’Ivoire.

In conclusion, despite the disruption of CDTi due to civil unrest (especially between 2002 and 2007), our results clearly show significant reductions in onchocerciasis prevalence in Côte d’Ivoire after the scale up of mass treatment with ivermectin. Our identification of potential foci with ongoing infection may help control programmes target intervention with ivermectin (with or without vector control) or more frequent ivermectin distribution in areas where infection persists despite adequate ivermectin coverage. Such focused efforts are likely to be very important in the late stages of the country’s onchocerciasis elimination programme. Although this study focused on Côte d’Ivoire, this approach may be useful for identifying endemic areas and targeting interventions to eliminate onchocerciasis in other African countries.

## Supporting information

S1 Fig Uncertainty maps shown as standard deviation at a resolution of 5km x 5 km. Plot on the left and right are standard deviation of model trained with data from 1975 to 1991 and 1992 to 2016, respectively.
(TIF)
