# Supplementary figures and images for: Progress towards onchocerciasis elimination in Côte d’Ivoire: A geospatial modelling study
Source: PLoS Negl Trop Dis. 2021 Feb 10;15(2):e0009091. doi: 10.1371/journal.pntd.0009091 (PMC7875389; doi:10.1371/journal.pntd.0009091)

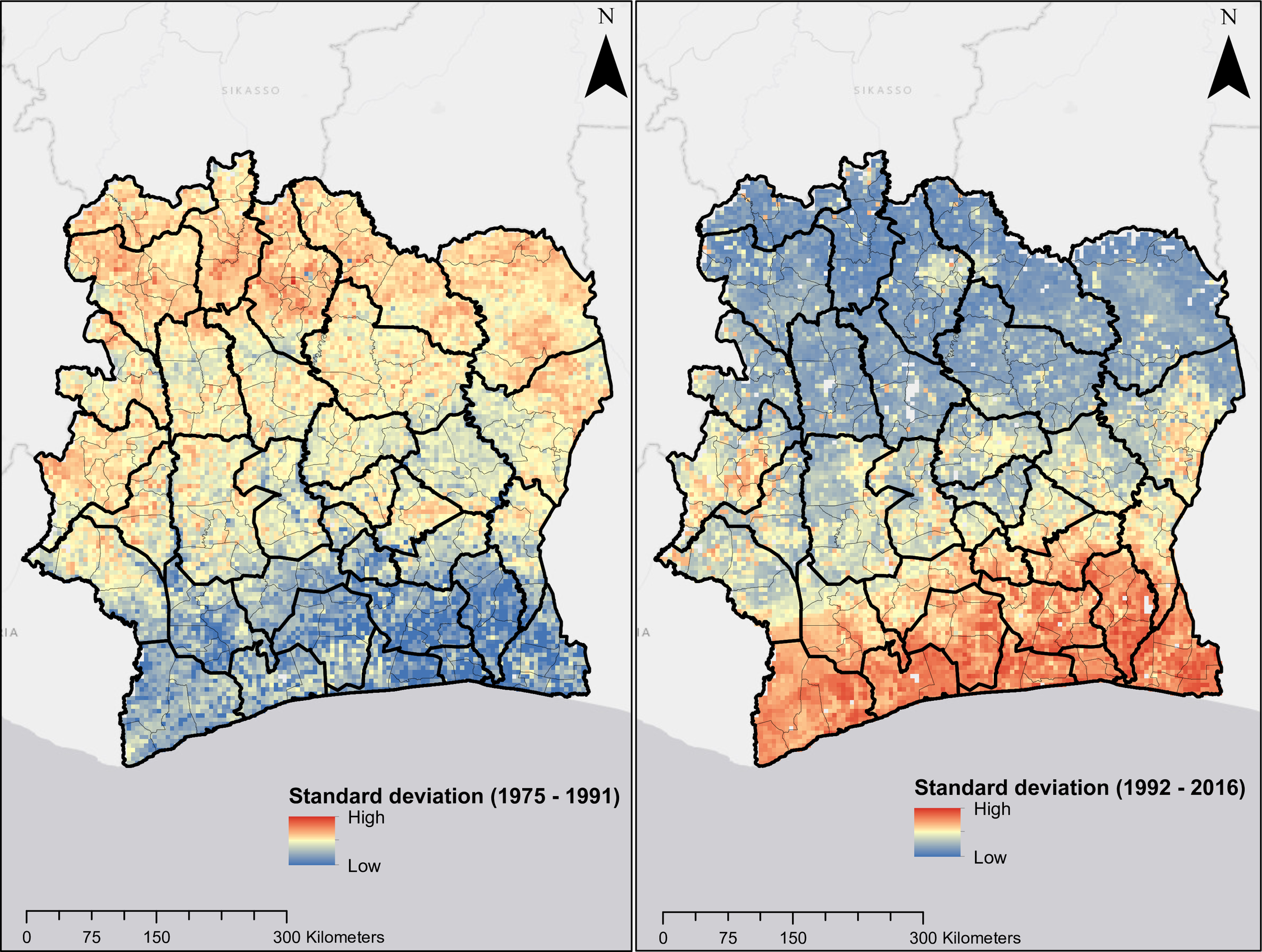

Supplement: S1 Fig — Plot on the left and right are standard deviation of model trained with data from 1975 to 1991 and 1992 to 2016, respectively. (TIF) [file pntd.0009091.s001.tif]
